# Development of a New Lab-on-Paper Microfluidics Platform Using Bi-Material Cantilever Actuators for ELISA on Paper

**DOI:** 10.3390/bios13030310

**Published:** 2023-02-23

**Authors:** Hojat Heidari-Bafroui, Ashutosh Kumar, Cameron Hahn, Nicholas Scholz, Amer Charbaji, Nassim Rahmani, Constantine Anagnostopoulos, Mohammad Faghri

**Affiliations:** Microfluidics Laboratory, Department of Mechanical, Industrial, and Systems Engineering, University of Rhode Island, 2 East Alumni Avenue, Kingston, RI 02881, USA

**Keywords:** colorimetric assay, paper-based devices, lab-on-paper, ELISA, point-of-care diagnostics, sensors, low-cost platforms

## Abstract

In this paper, we present a novel and cost-effective lab-on-paper microfluidics platform for performing ELISA autonomously, with no user intervention beyond adding the sample. The platform utilizes two Bi-Material Cantilever Valves placed in a specially designed housing. The integration of these valves in a specific channel network forms a complete fluidic logic circuit for performing ELISA on paper. The housing also incorporates an innovative reagent storage and release mechanism that minimizes variability in the volume of reagents released into the reagent pads. The platform design was optimized to minimize variance in the time of fluid wicking from the reagent pad, using a randomized design of experiment. The platform adheres to the World Health Organization’s ASSURED principles. The optimized design was used to conduct an ELISA for detecting rabbit immunoglobulin G (IgG) in a buffer, with a limit of detection of 2.27 ng/mL and a limit of quantification of 8.33 ng/mL. This represents a 58% improvement over previous ELISA methods for detecting rabbit IgG in buffer using portable microfluidic technology.

## 1. Introduction

Simple, easy-to-use, and inexpensive point-of-care diagnostic devices can help to enhance healthcare systems and their availability will help to reduce the spread of infectious diseases such as COVID-19. This is particularly important in resource-constrained settings, as well as in developing countries. One such tool is point-of-care testing (POCT), which allows for quick diagnostics that is accurate, sensitive, uses little sample and reagent volumes, and is very cost-effective. One of the most promising platforms for POC diagnostics are microfluidic paper-based analytical devices (μPADs) in which paper substrates are used to carry out the required analytical testing. These devices are in line with the directive set by the World Health Organization (WHO) for testing in resource-limited settings. The directive by WHO called for tests to be Affordable, Sensitive, Specific, User-friendly, Rapid and Robust, Equipment-free, and Deliverable, or ASSURED, in short [1].

Enzyme-linked immunosorbent assay (ELISA) is one of the most commonly used techniques to determine the presence of antigens in biological samples [2]. However, conventional ELISA is not suitable as a point-of-care test as it requires multiple sequential steps that necessitate user involvement. It is also a time-consuming technique which requires relatively large volumes of analytes and reagents, needs an expert to conduct, and necessitates an expensive plate reader [3]. Therefore, conventional ELISA does not meet the requirements for an ASSURED diagnostic test device. Several research groups have already developed microfluidic paper-based devices that can perform ELISA in order to make a portable device for complicated assays [4,5,6,7,8,9,10,11,12,13,14]. Unlike conventional ELISAs, performing an ELISA on a μPAD removes the operator’s technical skill requirements by eliminating the need to pipette and remove sets of reagents and buffers. In addition, this portable device offers faster reaction rates in comparison with conventional ELISAs [15].

One major challenge in fully automated microfluidic devices is the direct storage of reagents needed for conducting the test on the μPAD. Previously developed techniques used to release reagents, such as blister pouches, tended to be effective but complicated [16]. While the blisters themselves are not complicated to fabricate or fill with reagents, the required small puncture spikes are difficult to place in the μPAD or actuate during the test. Another technique developed for storing reagents on the device was “Micro-Perforated Vapor Barrier Films” [17], designed to seal a chamber with reagents inside and then puncture and release the reagents using pneumatic pressure. This technique is more complex in terms of needing very precise perforations on a film and the required use of a pressurized gas. This also complicates the overall geometry, resulting in a large amount of wasted reagent from frictional forces in the device. Another developed technique uses the bubbles found in bubble wrap to store the reagents [18,19,20,21]. This method involves injecting the reagent into the bubble, then resealing it when needed. While this method is much cheaper and simpler to use than the previous methods, it still presents the μPAD designer with several complications. The first is injecting the reagent fluid into the bubble, which required skill or a potentially expensive automated system, both of which increased the variability in the amount of fluid injected. Additionally, the release of the reagent fluid is highly dependent on the method of sealing the bubble, causing a relatively large variability in the volume of reagent fluid being released. Often, the reagent fluid is released with a “splashing” effect. This, in turn, leads to impactful losses due to the small internal volume of the bubbles, which is a problem as the accuracy is dependent on the volume of reagent fluid taking part in the actual test. In this paper, a new method for the on-device storing, releasing, and transporting of fluid reagents is introduced. This method, which is based on a micro centrifuge tube and a four-spring mechanism, is a more straightforward, simpler, and cost-effective solution that addresses the limitations of previous methods. The new method overcomes the challenges of controlling the volume of reagent fluid and reducing the variability of test results, which are crucial for ensuring the accuracy of the test results.

Automated microfluidic devices can perform complex multistep assays like an ELISA when they are embedded with micro valve components to provide proper reagent flow control and timing [22]. Originally, our proprietary lab-on-paper platform used fluidic valves consisting of a stack of hydrophobic and hydrophilic discs [7,23]. However, the hydrophobic compound used in these valves was found to chemically react with the reagents used for the detection. Additionally, the valves had long response times and required large volumes of actuation fluids. Various other valving systems for paper-based microfluidic devices have also been developed [24,25]. Some of them are simple [26,27] and self-contained [28,29], requiring little to no user intervention [23,30], whereas some user or electrical power source input is needed for other valve systems to function [31,32,33]. To remedy the shortcoming in the previously reported paper-based valves and actuator, our group have invented a paper-based cantilever (PBC) [34] and a bi-material cantilever (B-MaC) [35]. The B-MaC design is based around the mechanics of a two-material cantilever beam, consisting of a piece of paper on one side and a piece of tape on the other. As the fluid moves through the paper it expands [30]. Due to the resistance of the tape against the expansion it forces it to bend down in an arc in the direction of the tape and come into contact with the stationary component (SC) underneath [36]. The B-MaC is simple to build, uses small sample volumes for activation, and has a prompt response time.

The purpose of this study is to propose a platform that houses a dual cantilever paper-based microfluidic device that is capable of the autonomous detection of antigens in a sample solution. A test solution is applied to a sample pad in the center of the housing. Two micro centrifuge tubes filled with two reagent solutions are then placed into two separate chambers and punctured using a built-in needle spring system by pressing down on the capsules. Once the capsules are punctured, the reagent in each is released onto two paper strips. Based on the dual cantilever design of the system, the first fluid introduced to the detection zone is the sample, followed by the first reagent solution. This is due to the bending of the first cantilever. The first reagent washes out any sample remaining on the sample pad. The second reagent is then absorbed by the second cantilever due to activation by the first reagent. Therefore, this system serves as a benchtop platform that can be used for detecting chemicals or biomarkers of interest in the sample solution where many reagent fluids are engaged and sequential loading or timing delays are required. The applicability of the system is evaluated by detecting rabbit immunoglobulin G (IgG) in buffer by conducting an ELISA.

## 2. Materials and Methods

### 2.1. Material

The following items were utilized in the preparation of the dual paper-based cantilever test strip: Whatman filter paper grade 41 (GE Healthcare Whatman 41-1441866), Scotch^®^ Tape 600 (3M, St. Paul, MN, USA), and Whatman blotting paper (WHA10547922-Whatman^®^ gel blotting paper, Grade GB003). The paper-based valve layout was created with vector graphics software (CorelDraw Graphics Suite X6, Corel Corporation, Ottawa, ON, Canada) and cut out with a laser engraver (Epilog mini 40 W) in the cross-machine direction of the paper. To aid in visualizing fluid flow, a few drops of a food coloring (Wilton Icing Colors) were added to ASTM Type 1 deionized water (DI) (resistivity > 18 MΩ/cm, LabChem-LC267405). All 3D-printer items were printed by an Ultimaker S5 3D printer (Dynamism, Chicago, IL, USA) using eSUN PLA filament (Xiaogan, Hubei, China).

For conducting an ELISA, Rabbit IgG Isotype Control, SuperBlock™ T20 (TBS) Blocking Buffer, Invitrogen™ Phosphate-Buffered Saline (PBS), D-Trehalose dihydrate, Sucrose, and Glass Fiber Conjugate Pad Strips were purchased from Thermo Fisher Scientific (Waltham, MA, USA). Monoclonal Anti-Rabbit IgG (γ-chain specific) antibody produced in mouse was bought from Sigma-Aldrich (Saint Louis, MO, USA) and used as immobilized capture antibody on the Whatman^®^ nitrocellulose membrane filters purchased from the same vendor. Mouse monoclonal [SB62a] Anti-Rabbit IgG light chain labeled with Alkaline Phosphatase was provided from Abcom^®^ (Waltham, MA, USA). 5-bromo-4-chloro-3-indolyl phosphate (BCIP)/nitro blue tetrazolium (NBT) was purchased from Scripps Laboratories (San Diego, CA, USA) as the chromogenic substrate to the alkaline phosphatase label.

### 2.2. Bi-Material Cantilever

A cantilever actuator consists of a strip of filter paper partially laminated on one side with Scotch tape, having left a 2 mm distance from one end of the paper. The paper’s cellulose fibers experience hygro-expansion and expand when exposed to a fluid sample, but the length of the tape layer remains the same. As a result, the cantilever’s free end bends in the direction of the tape layer, while its fixed end stays connected to a sturdy support that prohibits movement. The cantilever bends and a fluidic channel at the stationary component (SC) can be opened or closed as a result of the paper and tape components reacting to the sample fluid differently. More details about B-MaC architecture or actuation can be found in [35,37]. Table 1 summarizes the optimal design aspects of the paper-based cantilever, based on an extensive parametric study previously conducted, to fabricate a B-MaC [35].

### 2.3. Dual Bi-Material Cantilevers System

**Figure 1:**
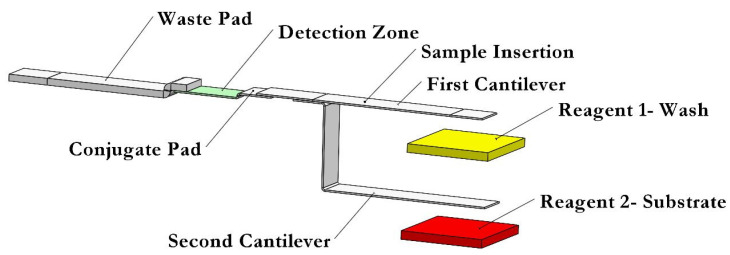
A schematic of the proposed dual B-MaCs assembly for performing an ELISA.

Figure 1 shows a schematic of the proposed dual B-MaC system designed to sequentially load two reagents and perform biomarker detection assays. In this system, the user only needs to apply a very small volume of the sample (5–10 µL) on the designated sample port to activate the first cantilever. Some of the sample fluid wicks toward the conjugate pad where the antigens in the sample fluid attach to the detection antibodies that are stored in the conjugate pad. These antibodies are tagged with an enzyme. The portion of the sample fluid flowing towards the cantilever causes the cantilever to bend downward and touch the reagent-1 pad, which is a wash solution. The contact causes the wash solution to flow by capillary action through the cantilever, push the sample solution back toward the conjugate pad where the antigens in the sample fluid attach to the detection antibodies and flow towards the detection site, where the conjugate consisting of antigen and detection antibody tagged with an enzyme is captured by the captured antibodies that were immobilized in the detection zone. Excess detection antibodies and enzymes are washed and flowed to the waste pad. A portion of reagent 1 (approximately 12 μL) flows towards the second cantilever to activate it. Reagent 2 then begins to flow through the second cantilever toward the detection zone and waste pad. The result is a sample solution and two reagents sequentially loaded onto the detection zone autonomously (aside from the initial application step).

### 2.4. Dual Bi-Material Cantilevers System for Performing an ELISA

**Figure 2:**
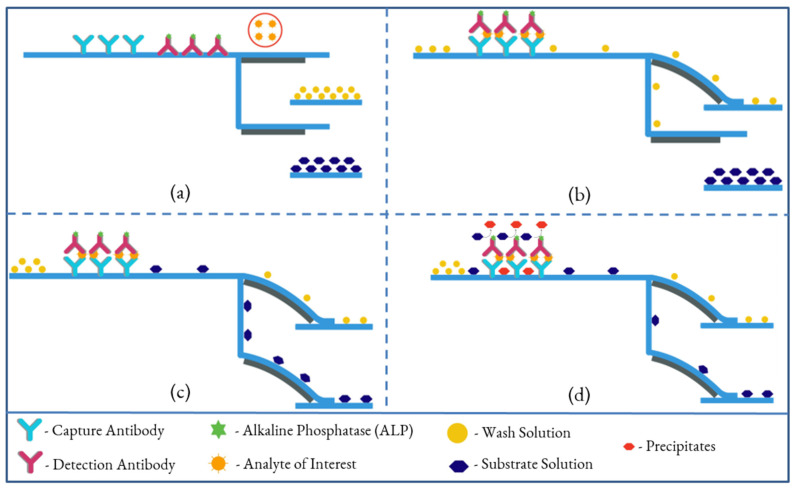
Illustration of performing an ELISA on a dual B-MaC assembly. (**a**): Activation of the first cantilever by introducing sample, (**b**): loading of wash solution onto the first cantilever, washing off any unabsorbed detection antibody from the conjugate pad, (**c**): activation of the second cantilever, initiating the loading of the substrate toward the detection zone, (**d**): colorimetric reaction and precipitation of reacted enzyme for quantifying the signal.

ELISA is widely used in biomedical analyses, including immunoassays, food industry assays for food allergens, and toxicological assays [38,39]. These assays are typically carried out in microtiter plates or small vials. ELISA on paper is a quantitative analytical method that shows antigen–antibody reactions through the color change obtained from an enzyme-linked conjugate and substrate interaction. Conventional ELISA, usually performed in 96-well plates, is quantitative and well-suited for large scale assays, but each assay requires large volumes (50–200 µL) of analyte and reagents. Additionally, the required incubation and blocking steps are long due to the diffusion of the reagents into the surface of the wells. The results are quantified using a plate reader, which is expensive lab equipment. Figure 2 shows the four essential steps for performing ELISA on paper without human interaction, other than sample insertion. The first cantilever is activated by the introduction of a sample to the system by an untrained user. The wash solution then loads onto the first cantilever, and washes off any unabsorbed detection antibodies tagged with enzyme from the conjugate pad, where detection antibodies are mobilized, to the waste pad. Simultaneously, some parts of the wash solution travel to the second cantilever through the delay channel to activate the second cantilever, initiating the loading of the substrate toward the detection zone. The substrate reacts with the enzymes, which are captured in the detection zone, and the reaction produces a red precipitate. The color intensity of the precipitate constitutes a detectable signal that can be quantified. The color intensity of the spot or line formed is related to the concentration of antigen present in the sample fluid.

### 2.5. Storing and Releasing Reagents Mechanism

**Figure 3:**
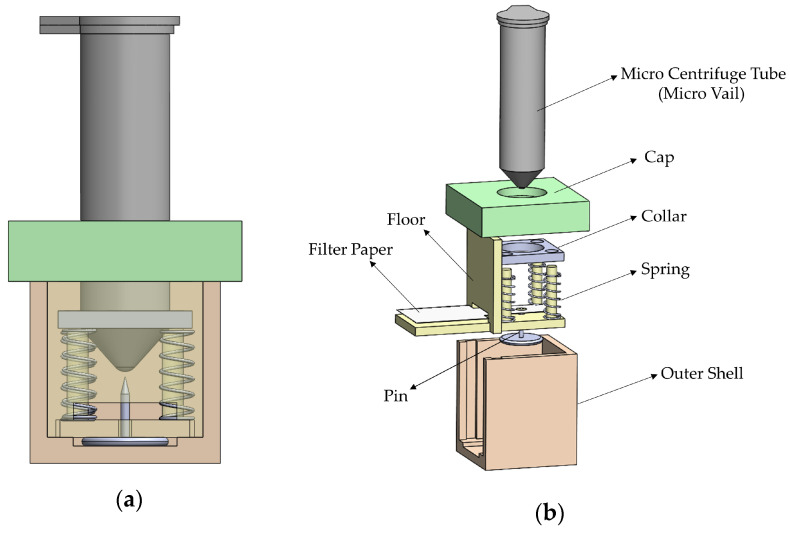
(**a**) A side view of the release mechanism. The inside of the outer shell appears by making the side part transparent. (**b**) An exploded view of the mechanism.

The release mechanism as shown in Figure 3 is composed of 11 parts: the outer shell, the floor, a pin, the micro centrifuge tube (including the collar), the cap, a strip of wicking paper, and four springs. The operation of the device begins with the micro centrifuge tube, full of reagent and with the collar attached to it. They rest inside the outer shell, with the collar resting on the springs. To activate, the micro centrifuge tube is pushed down to pierce the film and begin releasing the reagent. Finally, removing the cap of the tube allows the reagent to flow onto the wicking paper and onto the testing chamber. The goal of the device was not only to release the reagents when the operator wants to, but also to be inexpensive, consistent, and provide sealed on-device reagent storage.

The outer shell, the floor, the cap, and the collar were all 3D printed. The outer shell has a slot for the floor to be inserted, with enough room for the pin. The pin used required a gap of 2 mm for its bottom. The walls, the floor, the cap, and the collar are all 2 mm in thickness, allowing for the housing to be rigid, printable on most 3D printers, and created without wasting unnecessary plastic filament material. The height of the device allows for easier use by the operator, while still maintaining the desired height for both the tube to be aligned and the collar to remain fully in contact along the rails and springs. The floor consists of one of the outer shell walls, which allows for the assembly of the device, and four cylindrical rails, each being 3 mm in diameter. The collar is aligned on the rails with the springs in between the collar and the floor.

The micro centrifuge tube was drilled using a 3 mm diameter drill bit through its bottom and then covered with a film. This is accomplished by applying cling wrap around the bottom of the tube, with a tape sealing the wrap to the outer wall of the tube. The collar is then inserted around the tube and held in place by friction.

The pin used was an ordinary flat-headed thumbtack, the base of which is 1 mm thick, and the length from the base to the point is 5 mm. The spring’s dimensions are 0.3 mm thick with a free height of 10 mm, and an outer diameter of 4 mm. The wicking paper is Whatman Grade 41 paper and is 5 mm wide.

### 2.6. Platform Housing

**Figure 4:**
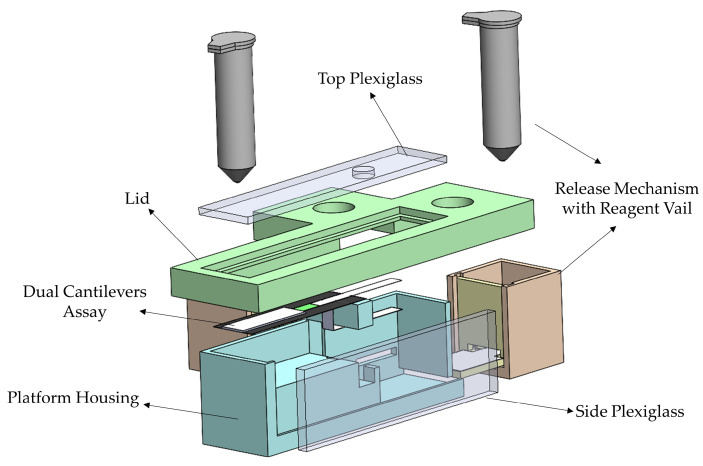
An exploded view of the proposed platform.

**Figure 5:**
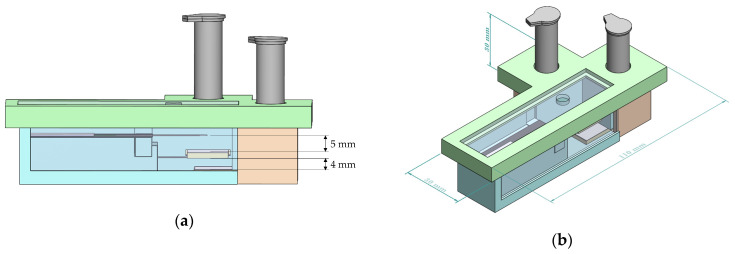
(**a**) A side view of the platform. (**b**) An isotropic view with dimensions of the platform.

The platform housing, shown in Figure 4, was designed to accommodate the dual bi-material cantilever system. Housing of the dual B-MaC is essential to carry out point of care testing with unaffected environmental and external conditions. The housing also aids the user to minimize the operational error associated with using the device for biological or chemical detection. The initial design of the proposed platform housing consisted of two release mechanisms (discussed in the above sections) with reagent vials, dual cantilever assembly assay, a securing lid, and two plexiglass windows. Loading of multiple reagents is a predominant characteristic of biological detection; this housing platform aids dual cantilever systems in the loading and detection of biological assays with two reagents. This is achieved by providing a securing lid for the platform, which helps to eliminate the interaction of the dual assembly with external conditions and provides an isolated space to carry out the required test. Figure 5a illustrates the critical design specifications essential for the functionality of the dual cantilever design. To prevent unintentional activation of the B-MaCs under higher humidity conditions of the platform due to the release of reagents to the system, 5 mm and 4 mm gaps are provided between the cantilever and the reagent pads. Previous extensive experimental testing has been carried out to study the effect of humidity and temperature on the functionality of B-MaC, indicating that a minimum distance of 4 mm is required between the B-MaC and the stationary component [35]. The proposed platform geometrical parameters highlighted in Figure 5b give a scale for the device.

### 2.7. Conjugate Pad

The glass fiber conjugate pads (8 × 5 mm^2^) were prepared using a SuperBlock T20 (TBS), sucrose, trehalose, and mouse monoclonal anti-Rabbit IgG light chain labeled with alkaline phosphatase as the detection antibody tagged with an enzyme. We followed the same preparation steps and sugar concentrations used in a previous study carried out in our lab [7].

First, 15 μL of SuperBlock containing 20% total sugar, composed of 10% trehalose and 10% sucrose, was pipetted onto the glass fiber. The coated glass fibers were then dried in an oven at 37 °C for 60 min. Once that was completed, 15 μL of a solution containing 20 μg/mL of the detection antibody in SuperBlock and 20% total sugar of the same composition as the previous solution was pipetted onto the pads. The pads were then put into the oven at 37 °C once again for 60 min.

### 2.8. Assay Preparation

For preparing an assay, first a 15 × 35 mm^2^ strip of backing card (MIBA-010, DCNovations, Carlsbad, CA, USA) was cut. The backing card supports the devices built on it while causing as little interference as possible, a feature of its pressure-sensitivity and non-reactive acrylic adhesive features [40]. Then, a strip of nitrocellulose, 5 mm wide by 15 mm long, was stuck on the center of the backing card strip. A total of 0.6 μL of the capture antibody at a concentration of 1.8 mg/mL, in increments of 0.2 μL, was dispensed near the center of the nitrocellulose membrane with a 10 min waiting time between each drop application, while making sure that the drops were all deposited on top of one another. The volume and concentration of the capture antibody have been previously optimized in [7]. The strip was dried overnight in a dry box at room temperature. Then, 25 μL of the SuperBlock blocking buffer solution was pipette twice on the nitrocellulose in order to block the remaining binding surface of nitrocellulose and to improve the signal-to-noise ratio of the assay. The strip was put in a covered Petri dish to be dried for an hour. Finally, the assay was assembled according to the schematic diagram shown in Figure 1 by using a 15 × 15 mm^2^ strip of Whatman blotting paper (WHA10547922-Whatman^®^ gel blotting paper, Grade GB003) as a waste pad, the prepared conjugate pad mentioned in Section 2.6, and two strips of bi-material cantilevers discussed in Section 2.2.

### 2.9. Assay Procedure

**Figure 6:**
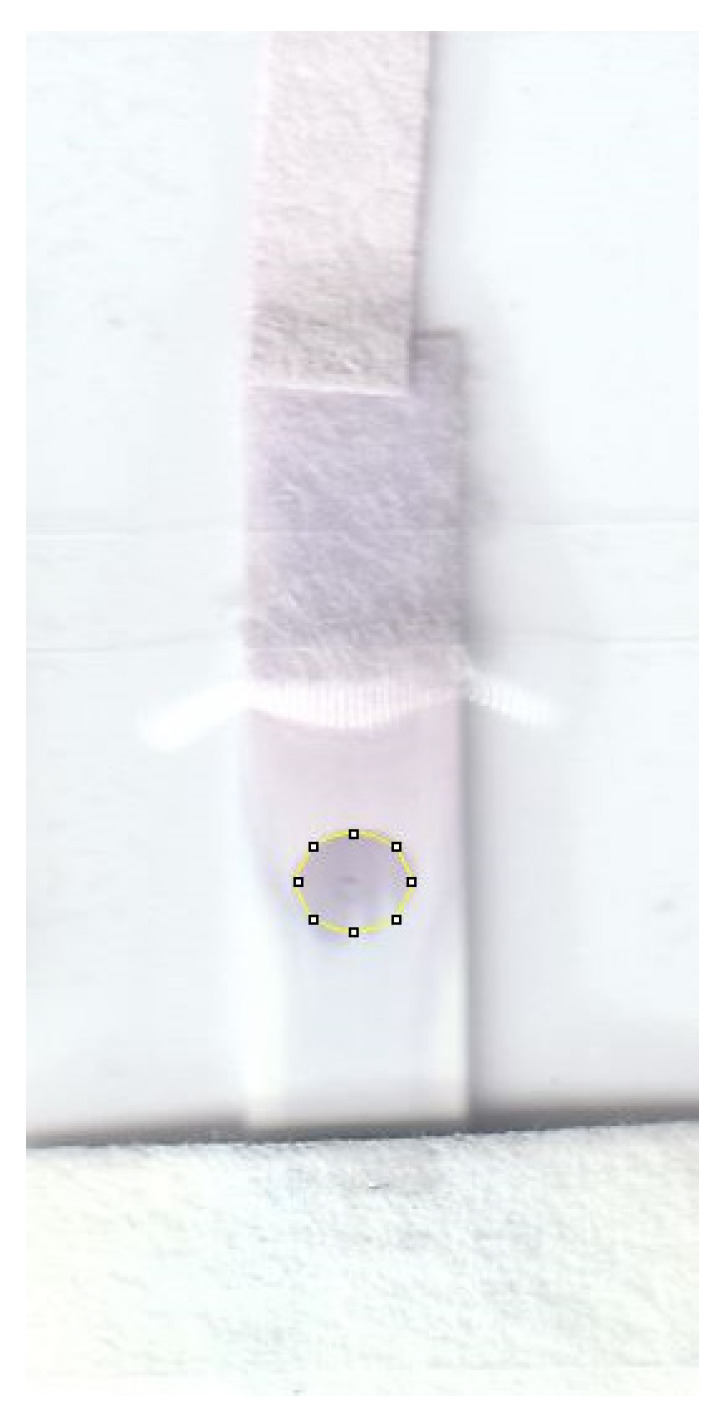
Detection zones showing the color that forms in the nitrocellulose. The region of interest, depicted as a yellow circle with a diameter of approximately 2 mm, is used to determine the color intensity by using ImageJ software.

Two micro vials with a 3 mm hole in their tip and sealed by a piece of tape are filled with 250 μL of the reagents of interest. The user only needs to tear the platform plastic sealing and insert the vials into the designated area for the reagents. Then, by pressing the vials to the end and opening the cap of vials, the reagents are loaded onto the filter papers underneath the vials. Afterwards, the test commences by applying 10 μL of the sample from the designed entrance on the plexiglass on top of the device. Upon completion of the test, the assay is extracted from the platform by opening the lid. The assay is then scanned using a Canon TS6020 desktop scanner with a scanning resolution of 600 DPI. The gray intensity of the detection zone is quantified by utilizing ImageJ software. It is worth noting that the platform can be integrated with a smartphone-controlled portable optical reader, thereby making the device completely portable [41]. The detection zone for the rabbit IgG experiments is located in the central region of the nitrocellulose membrane, where the capture antibodies have been immobilized. The region of interest used in this study to measure the color intensity by using ImageJ is depicted in Figure 6.

## 3. Results and Discussion

### 3.1. Hole Size of Release Mechanism

**Figure 7:**
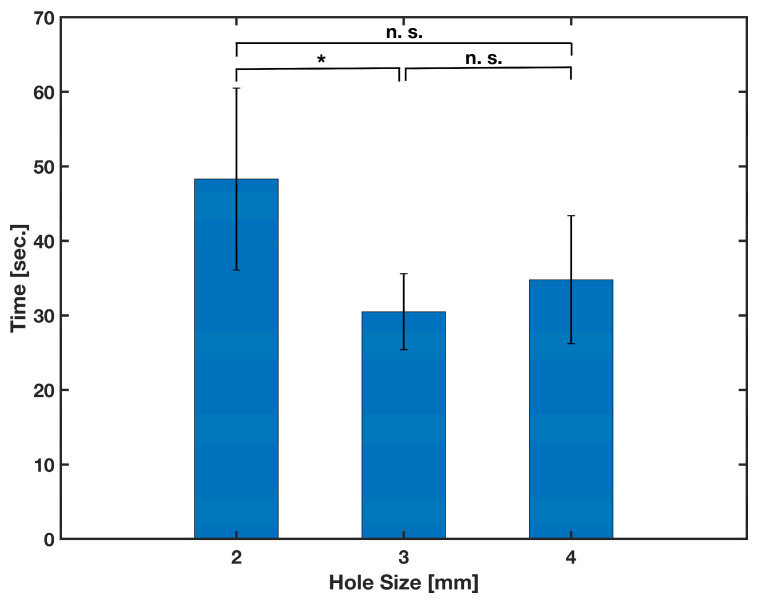
Effect of the vials’ hole size on the time of wicking of the reagent into the loading paper pad. Mean values were obtained from five different samples (*n* = 5) per condition. The error bars represent the standard deviation. Two-sample *t*-test was performed on the data (* *p*-value < 0.05 and n. s. stands for not significant).

The first parameter that needs optimization to produce a secure releasing reagent onto the loading paper is the hole size of the tip of the micro vials. A release mechanism housing with an extended track was 3D-printed. The housing was identical to the one in the full experiment at the time. However, it had an extended track that was around 10 cm long from the opening; a ruler was laid next to it for reference. Hole sizes from 1 mm to 5 mm in increments of 1 were selected to be tested. However, the 1 mm hole size was too small and caused the needle to close the hole when inserted, whereas the 5 mm hole size also failed since the boring of the hole reduced the overall length of the vial, making the needle less likely to come into contact with the vial seal. A completely randomized test order with five replicates was run for each 2, 3, and 4 mm hole size of the vials. The vials were filled with 500 μL of deionized water that was previously mixed with a few drops of red food coloring. The time between pressing down the vials and the traveling of the red fluid into the filter paper for 7 cm on the extended track was measured. The results are depicted in Figure 7, and were subjected to a one-way analysis of variance (ANOVA). Considering the calculated (5.353) and critical (*F*(2,12) = 9.408 at α = 0.05) *F*-values, a significant difference (*p*-value = 0.02) between the performance of the different hole sizes was statistically observed. Although the results obtained for 3 mm and 4 mm hole sizes were not statistically different (*p*-value = 0.24 for two-tailed *t*-test), the 3 mm hole size was selected for further experiments due to the smaller data variation observed in testing.

### 3.2. Performance of the Platform with Food Color Reagents

**Figure 8:**
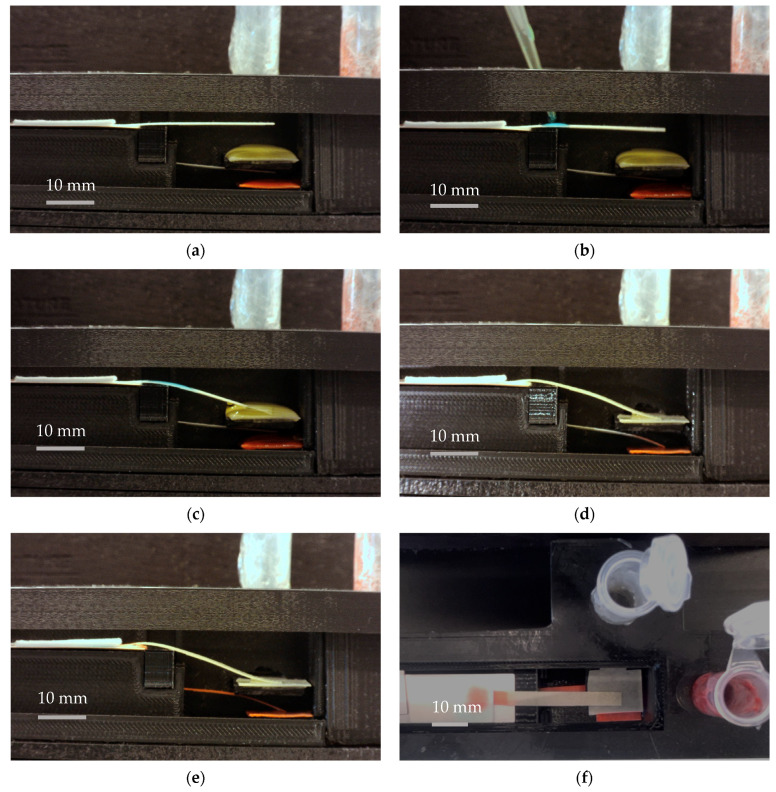
(**a**): Dual B-MaC assembly and positions with respect to the reagent pads (pre-saturated with food color reagents). (**b**): Sample fluid was introduced onto the top cantilever, initiating the loading of yellow reagent. (**c**): Yellow reagent delivered the sample to the conjugate pad and activated the bottom cantilever for the second reagent loading. (**d**): Initiation of the loading of the red reagent towards the detection zone. (**e**): The final results when all of the red reagent is transferred towards the waste pad after passing though the detection zone. The average time (*n* = 5) for each step is (**a**) t = 0, (**b**) t = 1 (s), (**c**) t = 4 ± 0.4 (s), (**d**) t = 260 ± 34 (s) and (**e**) t = 610 ± 81 (s). Subfigure (**f**) shows the top view of the device with the final results.

The proposed platform was used in testing with food color reagents to define critical design specifications of the device. Figure 8 illustrates different stages of functioning of a dual B-MaC assembly. For this purpose, two food color reagents, yellow and red as shown in Figure 8, were utilized. The performance of the platform with the reagents was tested with the following steps: step (a) shows the initial positions of the dual B-MaC assembly with respect to the reagent pads (pre-saturated with food color reagents). In step (b), sample fluid was introduced onto the top cantilever, initiating the loading of yellow reagent. In step (c), yellow reagent primarily helped in delivering the sample to the conjugate pad and activating the bottom cantilever for reagent loading, as shown in step (d), initiating the loading of red reagent towards the detection zone. During this process, the yellow reagent utilized for the activation of the second cantilever was transferred back towards the detection zone. Step (e) shows the final results when all the red reagent was transferred towards the waste pad after passing though the detection zone. Figure 8 illustrates these steps at an average (*n* = 5) time of 0 (s), 1 (s), 3 ± 0.4 (s), 260 ± 34 (s), and 610 ± 81 (s), respectively.

### 3.3. Shelf Life of Conjugate Pad

**Figure 9:**
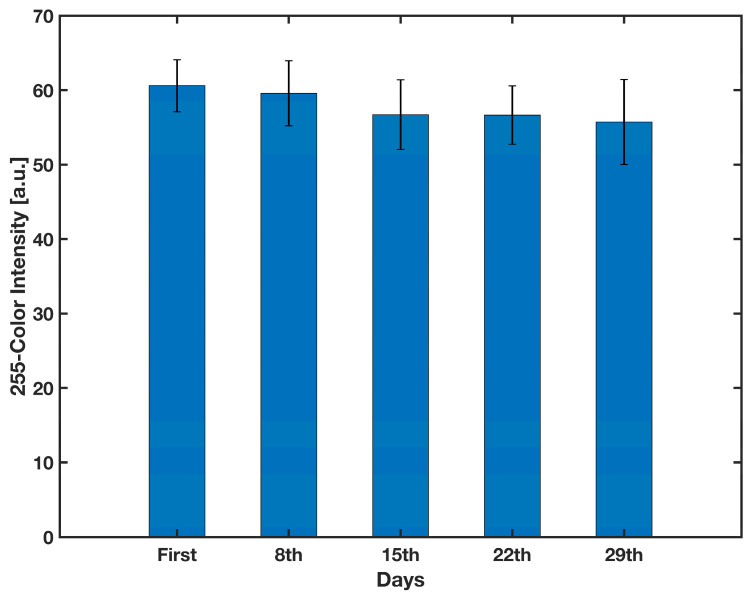
Effect of time on the color intensity formed on the detection zone. *n* = 5 and the error bars represent the standard deviation.

For the shelf-life experiment, 25 conjugate pads were simultaneously created using the method described in Section 2.6. Once every seven days, five conjugate pads were applied to a test strip consisting of a backing card, two pieces of filter paper with the dimensions of 10 × 5 mm^2^, the sample pad and the detection zone, put before and after the conjugate pad, respectively, and finally a waste pad of 20 × 5 mm^2^. These components were arranged to conduct a lateral flow test. In order to simulate the release of the detection antibody into the detection zone in the actual device, and also to study the functionality of the conjugate pad over time, 30 μL of DI water was first applied to the sample pad. After 30 min, the same amount of substrate was applied to the sample pad. The amount of labeled detection antibodies released from the conjugate pad and reaching to the detection zone directly correlates with the strength of the developing signal. Therefore, after 30 min the detection zones were scanned and the gray color intensity was measured using ImageJ. As shown in Figure 9, a one-way ANOVA reveals that there was not a statistically significant difference in color intensity of the detection zone between any of the groups (*F*(4,20) = 3.24, *F*-value = 1.535, *p*-value = 0.23). As a result, the conjugate pad can be stable for at least one month when stored in room conditions.

### 3.4. Rabbit IgG Detection

**Figure 10:**
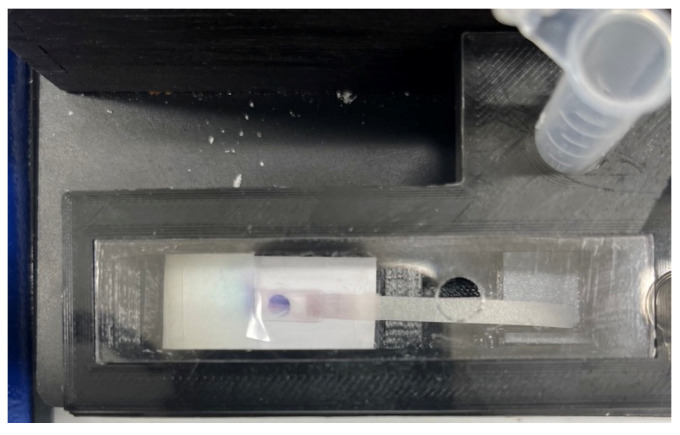
A signal formed in the detection zone for detection of 50 ng/mL of rabbit IgG in buffer.

**Figure 11:**
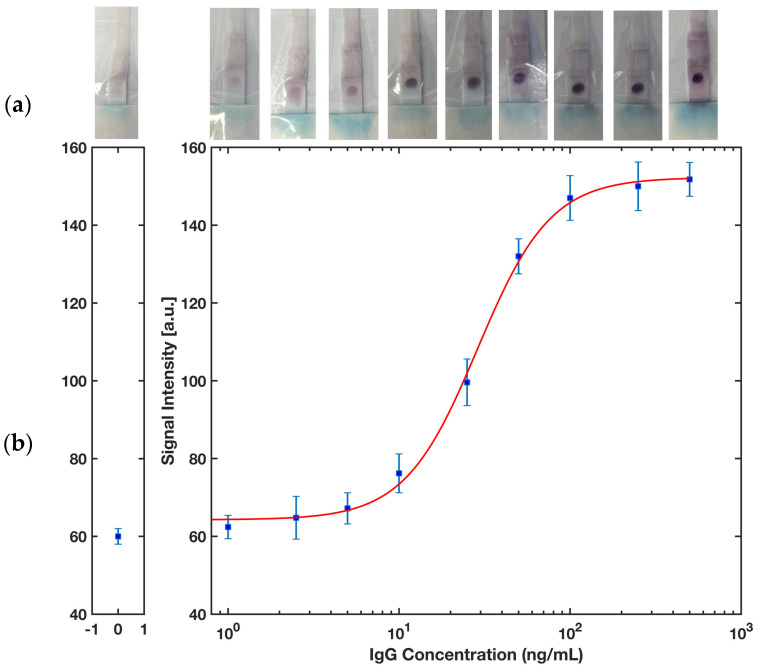
(**a**) A color chart showing the progress of color formation by increasing the rabbit IgG concentration in the sample. (**b**) A sigmoidal calibration plot of the intensity signal developed in the test zone versus the concentration of rabbit IgG in the sample. The error bars represent the standard deviation for *n* = 3.

Rabbit IgG in buffer was selected as a proof of concept of the ELISA protocol to detect an antigen of interest in the sample by the proposed platform. Figure 10 represents a colorimetric signal developed on the detection zone where the capture antibodies were immobilized. A series of rabbit IgG in SuperBlock Buffer solutions with concentrations 1, 2.5, 5, 10, 25, 50, 100, 250, and 500 ng/mL were prepared to run a completely randomized test order with three replicates for each concentration of sample. A calibration curve using MATLAB (Figure 11b) for computer-based quantitative detection and a color readout scale (Figure 11a) for qualitative naked-eye detection were generated as a result of this. Naked eye qualitative detection, however, has some limitations, including the potential for false positive results unless the reference strip shows a detectable difference in contrast [42]. To mitigate this issue, we have incorporated the use of a calibration reference strip (Figure 11a) into our detection process. The reference strip serves as a visual aid, providing a standard against which to compare the test results and ensuring that any changes in the contrast are accurately reflected. The use of a calibration reference strip helps to minimize the risk of false positive results and improves the reliability of the naked eye qualitative detection method.

A customized MATLAB code was developed to fit a sigmoidal curve in the form of a four-parameter logistic equation, as shown in Equation (1).

(1) $$y=d+\frac{a-d}{\left[1+{\left(\frac{x}{c}\right)}^{b}\right]}$$

where *y* is the dose–response; *x* is the arithmetic dose and *a* = 64.26, *b* = 2.03, *c* = 28.79 and *d* = 152.3 are parameters experimentally calculated. Next, the limits of detection (LOD) and quantification (LOQ) were calculated based on Equations (2) and (3) using the symbolic math toolbox in MATLAB.

(2) $$y_{lod}=y_{blank}+3\sigma_{blank}$$

(3) $$y_{loq}=y_{blank}+10\sigma_{blank}$$

where *y_blank_* and *σ_blank_* are the mean intensity and the standard deviation of the blank, i.e., no rabbit IgG in the sample, respectively. The limit of detection was calculated as low as 2.27 ng/mL and the limit of quantification was estimated as 8.33 ng/mL. Considering the molecular weight of 150 kDa for rabbit IgG [43], and that only 10 μL of sample was used for performing the test, the detection limit can be calculated to a molar concentration of 15.1 pM or a molar mass of 0.2 fm. To evaluate the reproducibility of the device, a test was conducted using 50 ng/mL rabbit IgG with five replicates and the relative standard deviation (RSD) value was found to be 3.4%, indicating a highly reproducible performance.

### 3.5. Comparison of Results with Those in the Literature

The limit of detection achieved here is superior to what was previously reported for the detection of rabbit IgG with paper-based ELISA platforms. The first paper-based ELISA was introduced by the Whitesides group [31], and required a small drop (2 μL) of sample. However, the device was not a fully autonomous platform as it required the user to move the strip manually through the device at specific times. In addition, the sensitivity of the method (LOD = 330 pM) was not as high as has been shown here. Gerbers et al. [7] demonstrated that complex diagnostic procedures can be performed autonomously in a new lateral flow device by incorporating an ELISA and rabbit IgG as a model analyte. They improved the sensitivity of a paper-based ELISA to a limit of detection of 36.7 pM for rabbit IgG test. However, long response times for changing the hydrophobicity of a multilayered structure using a surfactant to activate valves in their system, and a large volume of sample (130 μL), were device deficiencies. Qin et al. [38] introduced an easy-to-fabricate nitrocellulose (NC) paper plate for ELISA that, when combined with a desktop scanner, offered a greater protein immobilization efficiency than conventional cellulose paper-based ELISA devices. Although the reported NC paper plate needs a small amount of sample (5 μL), their method is not an autonomous technique and an expert user should follow a multistep protocol to detect the concentration of antigen in the sample. Finally, Kuo et al. [44] proposed a 3D folding ELISA device to detect different concentrations of rabbit IgG. They designed four wing-shaped zones for pre-loading reagents, and a center space was set aside as a testing zone for loading samples. The limit of detection of the IgG system using the device was 201 pM, while performing a test still requires the user to fold the different reagent zones into contact with the test zone by following a particular procedure. For further assessment, Table 2 provides a comparison between the sample volume, autonomous feature, signal detection method, and results achieved by the paper-based ELISA platform reported in this study and those obtained with paper-based ELISA devices previously presented.

## 4. Conclusions

In this study, a new, simple, and inexpensive platform that incorporates two Bi-Material Cantilever Valves that are placed in an especially designed housing was developed. The housing contains an especially designed reagent storage and release system that reduced the variability commonly observed with previously utilized storage mechanisms. The developed platform follows the ASSURED principles developed by the World Health Organization for point of care diagnostic testing and allowed for the detection of rabbit IgG in buffer using ELISA. The ELISA conducted in this study using this platform was fully autonomous and only required the user to place the sample at the start of the test. The limit of detection and quantification obtained were 2.27 ng/mL and 8.33 ng/mL, respectively. This accounts for more than a 58% improvement on what has been previously realized for the detection of rabbit IgG in buffer by ELISA using portable microfluidic technology.

## Figures and Tables

**Table 1:** Summary of optimal geometric and material parameters, along with corresponding activation time, for a B-MaC.

Parameter	Value
Paper type	Whatman filter paper grade 41
Paper direction	Cross-machine direction
Cantilever width	4 mm
Tape type	Scotch^®^ tape 600
Sample volume	8–12 µL
Activation time	3.1 ± 0.4 s

**Table 2:** Comparison of testing conditions and results attained by the paper-based ELISA platform presented in this study with previously reported devices.

Device	Sample Size (μL)	Autonomously	Signal Detection	LOD (pM)	Ref.
Movable paper-based strip	2	No	Low-cost desktop scanner	330	[31]
3D lateral flow device	130	Yes	Low-cost desktop scanner	36.7	[7]
Nitrocellulose paper-based multi-well plate	5	No	Low-cost desktop scanner	59.2	[38]
3D folding paper-based device	3	No	Low-cost desktop scanner	201	[44]
Standard plate-based ELISA	100	No	Expensive microplate reader	0.3	[44]
Dual B-MaCs platform	10	Yes	Low-cost desktop scanner	15.1	This work

## Data Availability

Data are contained within the article. Additional data not presented in this article are available on request from the corresponding author.
